# A Rapid and Reliable Absorbance Assay to Identify Drug–Drug Interactions with Thiopurine Drugs

**DOI:** 10.3390/metabo14120715

**Published:** 2024-12-19

**Authors:** Drake A. Russell, Carson Stafford, Rheem A. Totah

**Affiliations:** Department of Medicinal Chemistry, University of Washington, Seattle, WA 98195, USA; cstaff02@uw.edu (C.S.); rtotah@uw.edu (R.A.T.)

**Keywords:** drug–drug interaction, thiopurine methyltransferase (TPMT), methylation, mercaptopurine, small molecule methyltransferase

## Abstract

Background: Thiopurine methyltransferase (TPMT) plays a crucial role in the detoxification of thiopurine drugs, including the antimetabolites azathioprine and 6-mercaptopurine (6-MP) used to treat autoimmune diseases and various cancers. These drugs interfere with DNA synthesis by inhibiting the production of purine-containing nucleotides, leading to the death of rapidly dividing cells. TPMT inactivates thiopurine drugs by methylating at the thiol group. The activity of TPMT can vary significantly between individuals, and its activity is impacted by co-administered drugs, altering the effectiveness and toxicity of thiopurine drugs. TPMT is inhibited by many drugs that are co-administered to treat symptoms associated with diseases treated with thiopurines. For example, aspirin and other anti-inflammatory drugs, including olsalazine, sulfasalazine, and balsalazide, inhibit TPMT. The impact of TPMT genotypes on its methylating activity is well defined, and genotyping patients to identify TPMT metabolizer status is common clinical practice. Unfortunately, there has been no concerted effort to comprehensively identify drugs on the market that impact TPMT activity. The inhibition of TPMT by co-administered drugs could in part be responsible for idiosyncratic toxicities associated with thiopurine drug therapy. Methods: Here, we report a facile approach to produce large quantities of recombinant TPMT and a high-throughput assay that utilizes the shift in absorbance due to the methylation of thiopurines to report on TPMT activity. Results and Conclusions: With purified TPMT on hand and the absorbance activity assay, we confirmed several compounds that inhibit TPMT, and the results were comparable to a mass spectral assay that measured 6-MP methylation. Understanding the impact of co-administered drugs on TPMT activity will improve the safety and efficacy of thiopurine-based treatment regimens.

## 1. Introduction

**Figure 1:**
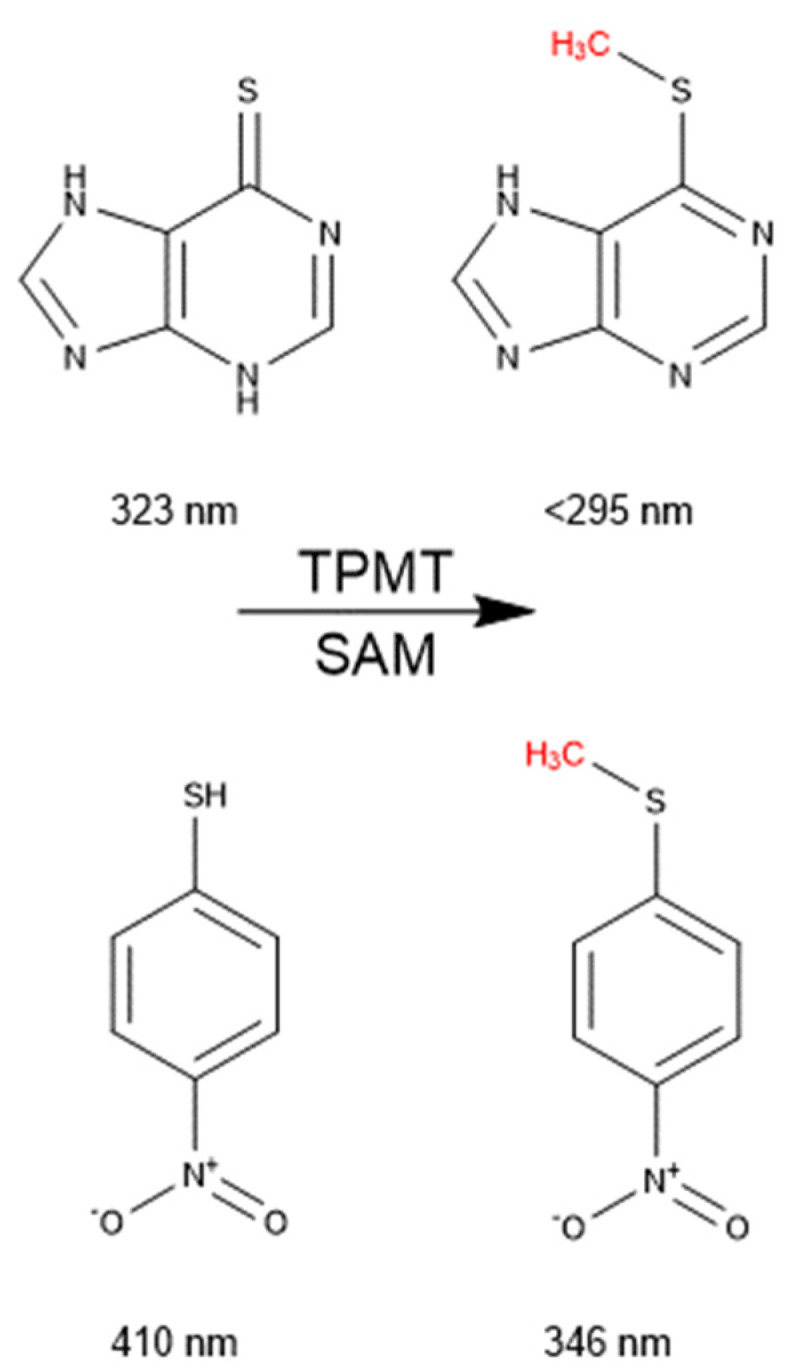
Reaction scheme of 6-MP and the methyl metabolite 6-MMP (**top**). Reaction scheme of 4-NBT and its methyl metabolite 4-NBMT (**bottom**). Each compound’s literature reported absorption maximum is written below its structure. The methyl group that TPMT adds to each molecule is in red.

Thiopurine methyltransferase is a small molecule, S-adenosyl-methionine (SAM)-dependent methyltransferase [1]. TPMT is essential for the metabolism of immunosuppressive and antileukemic prodrugs azathioprine, 6-mercaptopurine (6-MP) (Figure 1), and thioguanine [1,2,3]. These prodrugs are converted to thioguanosine monophosphate (TGMP) in vivo, which is an antimetabolite that mimics guanosine monophosphate [4]. Cells integrate TGMP into newly synthesized DNA instead of guanosine monophosphate. The accumulation of DNA with this foreign nucleotide is highly toxic, especially for fast-dividing cells [5,6]. Thiol methylation of thiopurines prevents their conversion to TGMP, inactivating them, and is the major route of metabolism for these compounds [7,8,9,10,11]. Because of the role TPMT plays in thiopurine metabolism, TGMP exposure is primarily dictated by TPMT activity [12].

The activity of TPMT is highly variable in a population [13]. Contributing to this variability are over 30 unique TPMT-variant alleles, some of which produce a TPMT enzyme with functionally low activity [14]. The effect that specific TPMT variants have on thiopurine metabolism is well understood. Organizations such as the Clinical Pharmacogenetics Implementation Consortium (CPIC) provide up-to-date guidance on metabolizer status based on the allele variant, and the United States Food and Drug Administration (FDA) recommends genetic testing for *TPMT* before initiating patients on thiopurine drugs [15,16].

A less understood contributor to TPMT activity is the effect of co-administered exogenous small molecules on TPMT’s ability to metabolize thiopurines. There are clinical examples of potential drug interactions leading to thiopurine toxicity. For example, benzoic acid derivatives potently inhibit TPMT [2,17]. Following therapeutic doses of aspirin, a popular over-the-counter pain medication, its metabolite, salicylic acid, circulates in plasma at concentrations that inhibit TPMT [2,17]. In a long-term clinical study of inflammatory bowel disease, 6-MP therapy was halted in 10% of patients because of adverse reactions [18]. More than half the people in that study were treated with the benzoic acid drug sulfasalazine. In another study, a patient with Crohn’s disease had to stop a 6-MP treatment because they experienced myelosuppression during treatment. This individual carried alleles of TPMT consistent with normal metabolizer status, but was also taking the benzoic acid drug olsalazine [19]. In a recent study, the formulation of 5-aminosalicylic acid (mesalazine), a known inhibitor of TPMT, was demonstrated to have a potential impact on the clinical effectiveness of thiopurines [20]. Although it is known that drug interactions with TPMT are possible, to our knowledge, there has been no concerted effort to screen approved medications taken concurrently with thiopurines for TPMT inhibition, and there currently is no FDA or CPIC guidance regarding the potential adverse effects resulting from this inhibition.

**Figure 2:**
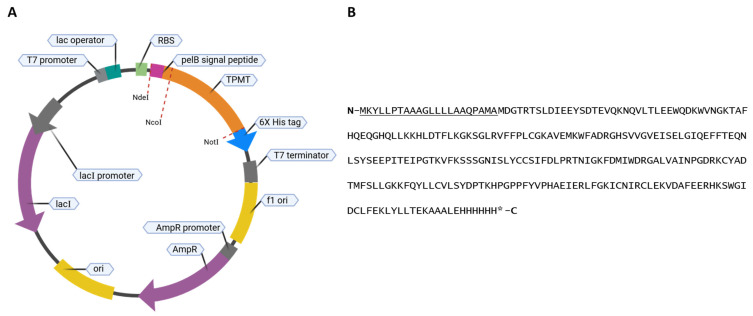
Map of pelB-TPMT-6XHis expression plasmid (**A**) and translated protein sequence (**B**). The pelB sequence is underlined. The estimated molecular weight of TPMT is 31.669 kDa with the pelB signal peptide and 29.458 kDa without. Weights calculated with Expasy molecular weight calculator [22,23,24,25].

Herein, we report two new tools that can be used to screen compounds easily and cost effectively for TPMT inhibition. We have developed a novel improved method for producing pure and highly active recombinant TPMT. This was accomplished by expressing TPMT in *E. coli* with a pelB *N*-terminal signal peptide (Figure 2). This signal peptide translocates TPMT to the bacteria cell’s periplasmic space. The periplasmic expression of proteins increases the yield of active protein by reducing proteolytic degradation, and provides a favorable environment for disulfide formation and proper protein folding [21]. Using this engineered plasmid, large quantities of TPMT were expressed with this recombinant system, and our purified TPMT was highly stable even after multiple rounds of freeze–thaw cycles. We then used the purified TPMT to develop a novel absorbance-based assay that can be used to screen compounds for TPMT inhibition. This assay monitors the change in absorbance wavelength of TPMT substrates as they are methylated. This is possible because several TPMT substrates, such as 6-MP and 4-nitrobenzenethiol (4-NBT), experience a large shift in absorbance when methylated by TPMT (Figure 1). We used this assay to screen for compounds that, when added to the methylation reactions, inhibit recombinant-purified TPMT. When applied to five different compounds, our absorbance assay identified TPMT inhibitors as effectively as a conventional multiple reaction monitoring mass spectrometry assay, but in a fraction of the time. The absorbance assay and new TPMT purification method are tools that can be used to expand the number of compounds screened for TPMT inhibition. An improved understanding of the factors contributing to variable TPMT activity across individuals will ultimately improve clinical practice when developing thiopurine-based treatment plans.

## 2. Materials and Methods

### 2.1. Chemicals, Materials, and Reagents

Activity assay substrates, reagents, cofactor, and inhibitors: balsalazide, sulfasalazine, and olsalazine were obtained from Cayman Chemicals (Ann Arbor, MI, USA); 4-nitrobenzenethiol was obtained from Tokyo Chemical Industry Co. (Portland, OR, USA); dopamine, 6-mercaptopurine, and 5-sulfasalycilic acid were obtained from Sigma-Aldrich (St. Louis, MO, USA); *S*-adenosyl-L-methionine was acquired from New England BioLabs (Ispwich, MA, USA); molecular-biology-grade water was obtained from Corning (Manassas, VA, USA); and DMSO was purchased from Fisher bioreagent (Fair Lawn, NJ, USA). Reagents and materials for *E. coli* culture, protein purification, and activity assay experiments: NaCl, KH_2_PO_4_, K_2_HPO_4_, imidazole, Thermo Scientific™ Halt™ Protease Inhibitor Cocktail, glycerol, CHAPS, and utra-15 10 K molecular weight cutoff centrifugal filter units were obtained from Fisher Scientific (Fair Lawn, NJ, USA); tryptone and yeast extract were purchased from gibco (Detroit, MI, USA); ampicillin sodium salt, deoxyribonuclease I, and lysozyme were obtained from Sigma Aldrich (St. Louis, MO, USA); and HisPur^TM^ Ni-NTA Resin was obtained from Thermo Scientific (Rockford, IL, USA). Reagents and materials for activity assay cleanup and downstream LC/MS analysis: Acetic Acid Optima LC/MS and Acetonitrile Optima LC/MS were acquired from Fischer Scientific (Fair Lawn, NJ, USA). Reagents and materials for SDS-page and Western blot analysis: PageRuler Plus Prestained Protein Ladder (10 to 250 kDa), GelCode™ Blue Safe Protein Stain, iBlot™ Nitrocellulose Transfer Stacks, and DTT were sourced from Fisher Scientific (Rockford, IL, USA); NuPAGE™ MOPS SDS Running Buffer (20×) and NuPAGE^TM^ 4–12% Bis-Tris Gel were acquired from Fisher Scientific (Carlsbad, CA, USA); TPMT rabbit PolyAb was obtained from proteintech^®^ (Rosemont, IL, USA); and IRDye^®^ 680RD goat anti-rabbit and 4× Loading Buffer were obtained from LI-COR (Lincoln, NE, USA). Reagents for plasmid editing and polymerase chain reaction: NcoI-HF, NcoI-HF, NEBNext Ultra II Q5 Master Mix, and Quick Ligase 2× Quick Ligase Reaction Buffer were all obtained from New England BioLabs (Ispwich, MA, USA).

### 2.2. Expression and Purification of TPMT

Wild-type TPMT was cloned in *E. coli* using the pET-22b (+) expression plasmid. The pET-22b (+) expression vector adds a pelB signal peptide to the *N* terminus of the inserted open reading frame (ORF) and a 6×histidine-tag at the *C*-terminus. The wild-type TPMT ORF was codon-optimized using Twist Bioscience’s ORF codon optimization tool. The ORF was synthesized by Twist Bioscience as a single DNA fragment. The ORF was inserted into the plasmid using NcoI and NotI restriction sites and general molecular biology techniques. The forward primer for the insertion of the TPMT ORF was 5′GCGATGGCCATGGATGGAACCCGTACCTCTCTT 3′, and the reverse primer was 5′ TCGAGTGCGGCCGCTTTTTCTGTTAACAGATACAGCTTCT 3′.

Expression plasmids were propagated using heat-shocked Stellar cells (Takara, Mountain View, CA, USA). Plasmids, validated by whole plasmid sequencing through Eurofins Genomics (Louisville, KY, USA), were used to transform competent LOBSTR-BL21 (DE3) *E. coli* (Kerafast, Winston-Salem, NC, USA) via heat shock. See Figure 2 for plasmid maps and translated protein sequences. Unless otherwise noted, *E. coli* cells were cultured in an orbital shaker at 250 rpm, 37 °C, and supplemented with 100 µg/mL ampicillin.

To express recombinant protein in LOBSTR-BL21 (DE3) cells, overnight cultures were added to ampicillin-containing terrific broth (TB) expression media at a ratio of 1:100. The cells were grown for 24 h. Cells were harvested by centrifuging at 4500× *g* for 15 min at 4 °C. The resulting cell pellet was stored at −80 °C. Frozen cell pellets were thawed on ice in a 4 °C cold cabinet prior to resuspension in lysis buffer (50 mM KPi pH 7.0, 20% glycerol, 150 mM NaCl, 10 mM CHAPS, EDTA-free 1× Halt Protease Inhibitor Cocktail) and supplemented with 100 µg/mL lysozyme (Sigma Aldrich, St. Louis, MO, USA, Ref: L-6876, Lot: 65H7025). The lysate was placed on a rocker at 4 °C until it became extremely viscous, then DNA Nuclease I (Sigma Aldrich, St. Louis, MO, USA, Ref: DN25-100MG, Lot: SLCB6648) was added to a final concentration of 100 µg/mL, and the lysate was placed on a rocker at 4 °C until it was no longer viscous. The lysate was then centrifuged at 48,000× *g* for 30 min at 4 °C, and the resulting supernatant was retained for subsequent purification steps.

The purification was performed using a vertical column hand-packed with 10 mL of His60 Ni Superflow Resin. The column was equilibrated with equilibration buffer (50 mM KPi pH 7.0, 20% glycerol, 150 mM NaCl, 10 mM CHAPS). Cell lysate supernatant was added to the column containing nickel resin, and it was recirculated across the column by a peristaltic pump overnight at 0.5 mL/min. The column was washed with 20 column volumes of His60 Ni wash I (50 mM KPi pH 7.0, 20% glycerol, 10 mM CHAPS, 300 mM NaCl, 50 mM imidazole). The column was then washed with 20 column volumes of His60 Ni wash II (50 mM KPi pH 7.0, 20% glycerol, 10 mM CHAPS, 150 mM NaCl, 50 mM imidazole). The column was then washed with 5 column volumes of transition wash (50 mM KPi pH 7.0, 20% glycerol, 1 mM CHAPs, 150 mM NaCl, 50 mM imidazole). The protein was eluted from the column with 5 column volumes of His60 Ni elution buffer (50 mM KPi pH 7.0, 20% glycerol, 1 mM CHAPS, 150 mM NaCl, 250 mM imidazole).

The purified protein was placed in a dialysis cassette with a 10 kDa molecular weight cutoff and immersed in dialysis buffer (50 mM KPi pH 7.0, 20% glycerol). The volume of dialysis buffer was 100× the volume of eluted TPMT. The protein was left in the dialysis buffer at 4 °C overnight with gentle agitation with a magnetic stir bar. The next day, the buffer was exchanged for fresh dialysis buffer, and the protein was incubated for another 4 h. This was repeated once more before the protein was transferred from the dialysis cassette to MilliporeSigma Amicon Ultra-15 10K molecule weight cutoff centrifugal filter units. The protein was concentrated, and the final protein concentration was determined by A280 measurement. Purified protein stocks were normalized to 1 mg/mL and stored at −80 °C for future use. Caution was exercised to ensure that the protein was kept at 4 °C during the entirety of the purification.

### 2.3. Purity Analysis of TPMT

All SDS-PAGE Coomassie stained or western blots were conducted with Invitrogen™ NuPAGE™ 4 to 12%, Bis-Tris, 1.0 mm, and Mini Protein Gels in the XCell SureLock Mini-Cell Electrophoresis system using PageRuler Plus Prestained Protein Ladder as a molecular weight marker. Gel electrophoresis was performed at room temperature and a constant 200 V. Total protein purity was determined with GelCode™ Blue Stain Reagent, a colloidal Coomassie dye. For western blot analysis, proteins were transferred to nitrocellulose membranes using the iBlot™ Gel Transfer Device. Primary antibody incubations were conducted overnight at 4 °C at a 1:500 dilution with rabbit anti-TPMT (Proteintech, Rosemont Il, Ref: 10682-1-AP, Lot: 00065222). The secondary incubation was performed at room temperature for 1 h with IRDye 680RD goat anti-rabbit secondary antibody at a 1:10,000 dilution (LiCor, Lincoln, NE, USA Ref: 926-68071, Lot: D11102-15). Western blots and Coomassie-stained gels were visualized with an Odyssey gel scanner, and blot images were analyzed using Image Studio Version 4.0 software.

### 2.4. High-Resolution Proteomic Analysis of Intact TPMT

In total, 20 µg of recombinant-purified TPMT was injected onto a qTOF SciEx 5600 mass spectrometer paired with a Waters ACQUITY LC and an Agilent PLRP-S 1000Å, 5 µm, 2.1 × 50 mm column, and 0.1% formic acid in water and 0.1% formic acid in acetonitrile as solvents A and B, respectively.

The elution of purified TPMT was achieved using the following gradient: The ratio of solvent A to solvent B was held at 90:10 from 0 to 1 min, then transitioned to 10:90 from 1 to 8 min with a gradient curve of 6. The ratio was then held at 10:90 from 8 to 9 min. The flow rate was set at a constant 0.3 mL/min. The instrument parameters were set to perform a continuous time-of-flight mass spec scan from 50 to 4000 Da. The collision energy was set to 5 eV, and the delustering potential was set to 120 V.

### 2.5. Absorbance Activity Assay Using Recombinant-Purified TPMT

To test the thiopurine methyltransferase activity of recombinant-purified TPMT, the enzyme was first diluted to 0.21 or 0.11 mg/mL with KPi reaction buffer (50 mM KPi, pH 7.0, 20% glycerol). A fraction of the diluted protein sample was boiled for 5 min. Diluted protein, 96 µL, was deposited in the appropriate wells of a 96-well Corning, black-walled, clear-bottomed, and half-area plate (Ref: 07200844). Substrates 6-MP or 4-NBT were added to the appropriate wells containing diluted TPMT. Both 6-MP and 4-NBT were dissolved in DMSO. For background vehicle control (VC) wells, DMSO was added instead. Finally, SAM was added to initiate the reaction. Water was added to the control samples that were not treated with SAM. The final reaction volume was 100 µL, and each reaction’s final DMSO content was 1%. The final concentration of 6-MP was 50 µM, the final concentration of 4-NBT was 200 µM, and the final concentration of SAM was 1 mM. The final concentration of TPMT treated with 6-MP was 0.20 mg/mL, and the final concentration of TPMT treated with 4-NBT was 0.10 mg/mL. All sample sets were performed in triplicate. After initiating the reaction with SAM, the 96-well plate was immediately placed in a Tecan Spark Multi-Mode Microplate Reader. Active protein samples containing 6-MP and SAM were scanned from 295 to 400 nm in 1 nm steps for 60 min, and active protein samples containing 4-NBT and SAM were scanned from 300 to 550 nm in 1 nm steps for 60 min. All boiled protein samples and samples not treated with SAM were scanned at the start of the incubation: time equals 0 min (T0), and at the end: time equals 60 min (T60).

### 2.6. Absorbance Inhibition Assay Using 4-NBT as a Probe Substrate

To determine if the absorbance assay utilizing 4-NBT as a probe substrate can be used to identify compounds that inhibit TPMT, the absorbance assay described previously was modified slightly. TPMT was diluted to 0.26 mg/mL, and then 95 µL of diluted protein was deposited in the appropriate wells of a 96-well Corning, black-walled, clear-bottomed, and half-area plate (Ref: 07200844). The pan-methyltransferase inhibitor S-adenosyl-homocysteine (SAH) was deposited into each well. The substrate 4-NBT was added to each well, and SAM was added to initiate the reaction. One set of samples was not treated with SAH, and another set was not treated with SAM or SAH. The final concentration of TPMT was 0.25 mg/mL, the final concentration of 4-NBT was 250 µM, the final concentration of SAH was 0, 150, 225, 300, 450, or 600 µM, and the final concentration of SAM was 500 µM. Note that SAH is a SAM-competitive inhibitor, so the concentration of SAM was dropped from a typical reaction concentration of 1 mM to 500 µM. All sample sets were performed in duplicate. After initiating the reaction, the 96-well plate was immediately placed in a Tecan Spark Multi-Mode Microplate Reader. Each sample was scanned from 300 to 550 nm in 1 nm increments for 60 min.

The absorbance assay was modified slightly to use low amounts of purified TPMT to be able to screen for potential inhibitors with minimum protein. For this modified inhibitor assay, the final concentration of 4-NBT was reduced to a final concentration of 200 µM, the final concentration of TPMT was reduced to 0.05 mg/mL, and the final concentration of SAM was at a typical methyltransferase reaction concentration of 1 mM. The final reaction volume of 100 µL was maintained. After initiating the reaction, the 96-well plate was immediately placed in a Tecan Spark Multi-Mode Microplate Reader, and each sample was scanned from 300 to 550 nm in 1 nm increments, once at T0 and once at T60. For time points between T0 and T60, each sample was scanned continuously at 411 nm.

### 2.7. Mass Spectrometry Activity Assay Validation Using Recombinant-Purified TPMT

Recombinant-purified TPMT was diluted into KPi reaction buffer (50 mM KPi, pH 7.0, 20% glycerol) to 0.12 mg/mL. A fraction of the diluted TPMT was reserved for boil control. The boil control samples were heated in boiling water for 5 min. A volume of 95 µL of either inactivated (boiled) or active TPMT was transferred to individual wells in a 350 µL 96-well plate; then, 6-MP was added, followed by a test inhibitor or vehicle control for samples not treated with an inactivator. The test inhibitor stocks contained 10% DMSO, so the vehicle control was water with 10% DMSO. The reaction was initiated by the addition of SAM. Samples without SAM received the same volume of water as a vehicle control. The final volume of the enzyme reaction was 100 µL. The final 6-MP concentration was 200 µM, the final test inhibitor concentration was 50 µM, and the final SAM concentration was 1 mM. Samples contained less than or equal to 1.1% DMSO. The plate was capped with a silicon mat and allowed to shake at 625 rpm for 5 min. The samples were then incubated for 1 h at 37 °C. After incubation, the reaction was quenched by adding 50 µL of each sample to 50 µL of ice-cold acetonitrile containing 40 µM of the internal standard (IS), D3-6-methyl mercaptopurine (D_3_-6-MMP), and 1% acetic acid. The samples were centrifuged at 4 °C and 16,000× *g* for 10 min to precipitate proteins and salts, and the supernatant was then transferred to a clean 96-well plate and analyzed by mass spec.

The samples were analyzed with a Waters TQS mass spectrometer paired with a Waters ACQUITY LC using a 2.1 × 50 mm Waters CORTECS UPLC 1.6 µm C18 column and 0.5% acetic acid in water and 0.5% acetic acid in acetonitrile as solvents A and B, respectively. The following gradient was used to elute 6-methyl mercaptopurine (6-MMP) and D_3_-6-MMP: The ratio of solvent A to solvent B was held at 95:5 from 0 to 1 min, then transitioned to 50:50 and was held at 50:50 from 1 to 2.5 min, from 2.5 min to 3 min the solvent ratio was held at 5:95, and, finally, the column was re-equilibrated with a solvent ratio of 95:5 solvent A to solvent B for half a min. The flow rate was held at 0.4 mL/min.

The mass transitions used to monitor for methyl metabolite 6-MMP and IS D_3_-6-MMP were 167.18 > 124.99 and 170.22 > 125.02, respectively; 6-MMP methylation was reported as the peak area ratio (PAR) of 6-MMP/D_3_-6-MMP.

### 2.8. Data Analysis

Unless otherwise noted, all experiments were conducted in triplicate, and all data are reported as the mean  ±  standard deviation. Statistical significance was determined by a two-tailed unpaired Student’s *t*-test with a threshold *p* value of 0.05. To calculate ΔAbs (411 nm) T0-T60, the absorbance at 411 nm taken at T0 was subtracted from the 411 nm absorbance taken at timepoint T60. The molecular weight of recombinant-purified TPMT was calculated with Expasy Molecular Weight Calculator, accessed at https://web.expasy.org/compute_pi/ (accessed on 5 August 2024).

## 3. Results

### 3.1. Expression of TPMT with an N-Terminal PelB Signal Peptide

**Figure 3:**
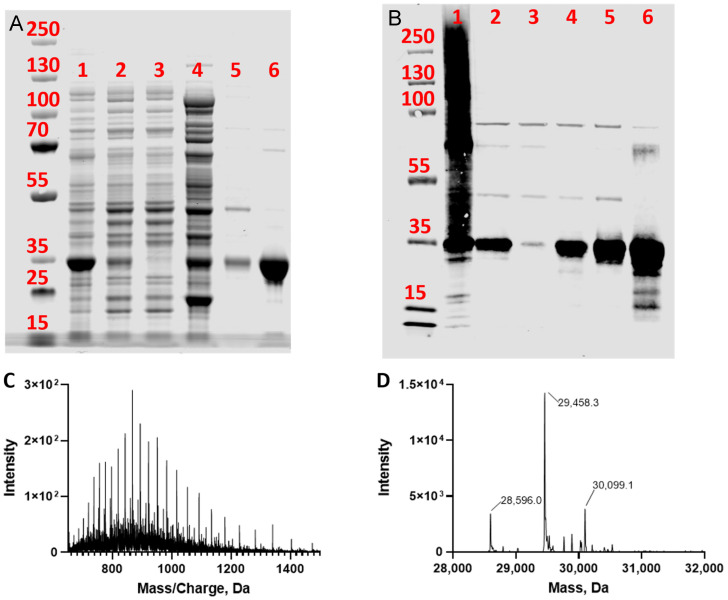
SDS-PAGE Coomassie stain of recombinant-purified TPMT with protein-normalized purification components (**A**). Anti-TPMT western blot (**B**). Lane order: homogenate (1), lysate (2), lysate post Ni column load (3), wash 1 (4), wash 2 (5), and eluent (6). Mass envelope of recombinant-purified TPMT (**C**) and deconvoluted exact mass (**D**). The molecular weight marker is the lane to the left of lane 1 in each blot, and the numbers in this lane indicate the molecular weight (kDa) of each band in it.

We inserted a TPMT open reading frame (ORF) into a pET-22b (+) expression vector and expressed the protein in *E. coli*. The pET-22b (+) expression vector adds a pelB periplasmic translocation sequence to the *N*-terminus of the inserted ORF and a 6xhistidine-tag to the *C*-terminus. The pelB signal peptide is removed as the protein is translocated to the periplasm [26]. The recombinant protein TPMT-6xHis has a molecular weight of 31.669 kDa with the pelB signal peptide and a molecular weight of 29.458 kDa once it is cleaved. We confirmed the successful purification of TPMT with a protein band visible at ~30 kDa by SDS-PAGE Coomassie stain (Figure 3A). A strong band at around 30 kDa is also visible on a western blot using an anti-TPMT-antibody (Figure 3B). Using SDS-PAGE analysis, there was a strong band visible around 30 kDa, which indicated that the purified TPMT had its pelB signal peptide removed during bacterial expression. The blots in Figure 3 were slightly overloaded with protein in their sixth lane. So, to confirm purity, we ran a second SDS-PAGE Coomassie stain with a lower amount of loaded protein. In this blot (Figure S1), we observed a faint band below the strong band ~30 kDa. Using intact proteomic analysis, we identified the deconvoluted mass for the purified TPMT of 29.458 kDa, consistent with the expected mass for TPMT without the pelB signal peptide. We also identified a protein with a deconvoluted mass of 28.596 kDa (Figure 3D), which could be related to the small band immediately below the TPMT band at ~30 kDa, although we were not able to verify the identity of this small contaminant protein. Our western blot, SDS-PAGE, and proteomic analyses confirmed that the recombinant TPMT was purified to near-homogeneity.

### 3.2. An Absorbance Assay to Determine the Activity of Purified TPMT

**Figure 4:**
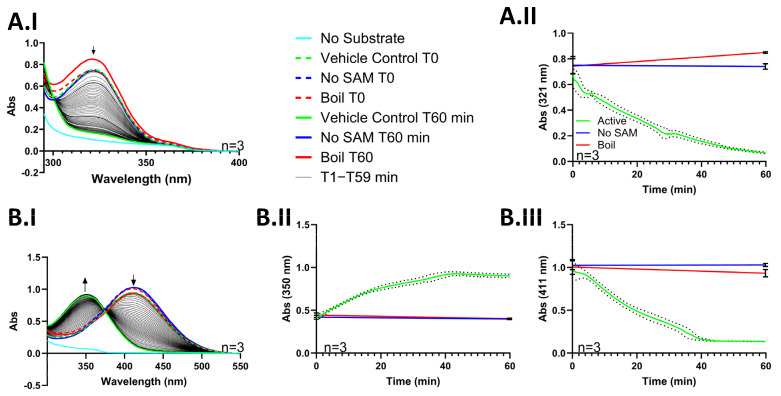
A scan from 295 nm to 400 nm (**A.I**) measured once every minute and the single wavelength absorbance at 321 nm (**A.II**) taken continuously during a 1 h incubation demonstrates the disappearance of 6-MP as it is converted to 6-MMP. A scan from 300 nm to 550 nm (**B.I**) taken once every minute, the single wavelength absorbance at 350 nm (**B.II**), and the single wavelength absorbance at 411 nm (**B.III**) continuously measured during a 1 h incubation demonstrate the disappearance of 4-NBT and the appearance of 4-NBMT. Data in (**A.I**,**B.I**) are presented as the mean. The arrows in (**A.I**,**B.I**) represent the direction of change in absorbance at the wavelengths indicated by each arrow. Data in (**A.II**,**B.II**,**B.III**) are presented as the mean ± S.D. The dotted lines in (**A.II**,**B.II**,**B.III**) indicate the SD for the “Active” treatment.

We determined that 6-MP has an absorbance maximum of 321 nm. This is in close alignment with the literature-reported value of 323 nm [27]. When 6-MP and SAM were incubated with purified TPMT, the reaction mixture’s absorbance at 321 nm decreased relative to time. The absorption maximum for 6-MP is 321 nm, but its methyl metabolite, 6-MMP, has an absorption maximum below 295 nm and does not appreciably absorb at 321 nm [27]. There was a decrease at 321 nm relative to time because the 6-MP in solution was becoming methylated. We confirmed that purified TPMT was active by monitoring 6-MP disappearance with a plate reader scanning from 295 nm to 400 nm for 1 h (Figure 4A.I). By monitoring absorbance change at 321 nm, we could record 6-MP disappearance in real-time during a TPMT and 6-MP methyl transfer reaction (Figure 4A.II). Within 60 min of initiating the enzyme reaction, the reaction mixture’s absorbance at 321 nm was reduced entirely to baseline.

The absorbance maximum of the TPMT substrate, 4-NBT [2], also changes when methylated. We determined that 4-NBT has an absorption maximum of 411 nm, but once methylated, its maximum shifts to 350 nm. This is in close alignment with the literature-reported values of 410 and 346 nm, respectively [28,29]. When 4-NBT and SAM were incubated with recombinant-purified TPMT, 4-NBT’s absorption maximum at 411 nm decreased with time, and 4-nitrobenzenemethylthiol’s (4-NBMT) absorption maximum at 350 nm increased accordingly (Figure 4B.I). Within 45 min of initiating the enzyme reaction, the absorbance at 411 nm was reduced entirely to baseline, and a new absorbance maximum at 350 nm appeared. A point between the 350 and 411 nm maxima at ~375 nm did not change in absorbance during the incubation. The clear isosbestic point at 375 nm confirms that only two species are observed within the 300–550 nm absorbance spectrum. After confirming that our recombinant-purified TPMT was active and that 4-NBT was an ideal probe substrate for an absorbance-based activity assay, we performed several freeze–thaw cycles on TPMT to determine the enzyme’s stability. Throughout five consecutive freeze–thaw cycles, there was no appreciable reduction in TPMT activity (Figure S2). These results indicate that 4-NBT is an excellent substrate to pair with an absorbance detection assay to report on the methylation activity of recombinant-purified TPMT.

### 3.3. Validation of 4-NBT Absorbance Assay for TPMT Inhibitor Screening

**Figure 5:**
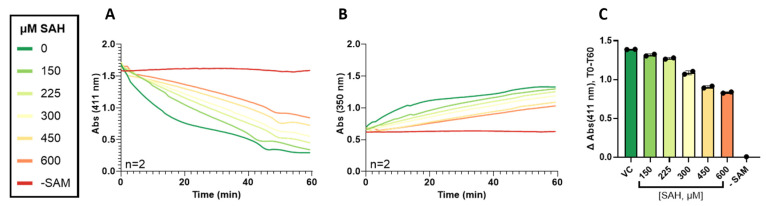
Real-time monitoring of 4-NBT disappearance (**A**) and 4-NBMT appearance (**B**) when TPMT is incubated with increasing concentrations of SAH. The difference in absorbance at 411 nm from T0 to T60 (**C**) demonstrates that, as the concentration of SAH increases, the amount of 4-NBT methylated in 60 min decreases. The black dots in C represent individual replicates, *n* = 2. Data in (**A**,**B**) are presented as the mean. Data in (**C**) are presented as the mean ± S.D.

Due to 4-NBT’s clear shift in absorbance maximum from 411 to 350 nm when methylated, and its relatively low reported K_m_ of 6.5 µM, we chose to use 4-NBT as a reporter substrate for TPMT activity. To confirm that an absorbance assay with 4-NBT as a reporter could be used to register TPMT inhibition, TPMT, 4-NBT, and SAM were incubated with various concentrations of SAM-dependent methyltransferase inhibitor SAH. As the concentration of SAH increased, the magnitude of absorbance change at 411 nm from T0 to T60 (∆Abs (411 nm)) decreased, and the absorbance at 350 nm at any given time during the reaction decreased with the concentration of SAH (Figure 5).

**Figure 6:**
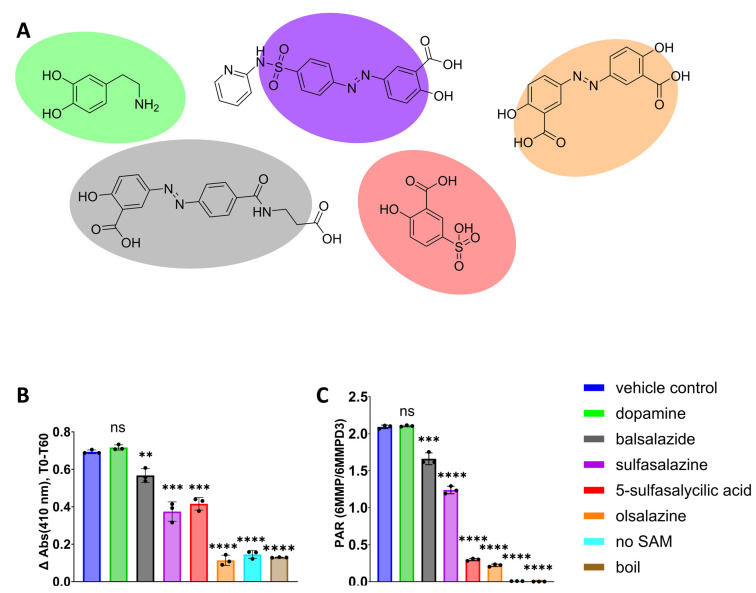
Test inhibitors (**A**). From left to right: dopamine, balsalazide, sulfasalazine, 5-sulfosalicylic acid, olsalazine. The difference in absorbance at 411 nm from T0 to T60 (**B**) in the presence of various test inactivators; 6-MP methylation by TPMT in the presence of different test inhibitors (**C**) determined by LC-MS-MS analysis. The black dots in B and C represent individual replicates, *n* = 3. Data are presented as the mean ± S.D. **** *p* < 0.0001; *** *p* < 0.001; ** *p* < 0.01.

After confirming that TPMT inhibition can be registered with the 4-NBT absorbance assay, we chose several test inhibitors (Figure 6A) to be included in a TPMT inhibitor screen. The test inhibitors, balsalazide, sulfasalazine, and olsalazine, had previously been identified as inhibitors of TPMT [30,31]. Benzoic acids are known to inhibit TPMT; 5-sulfosalicylic acid was chosen because it is a benzoic acid that has not yet been confirmed to inhibit TPMT [2]. Dopamine, known not to be a substrate of TPMT and not expected to inhibit TPMT [1], was included in the test set to control for the false-positive identification of TPMT inhibition. Each test compound was confirmed to not have an absorbance maximum that overlapped with 4-NBT’s (Figure S3).

Each test inhibitor (Figure 6A) was incubated with TPMT, 4-NBT, and SAM. The final concentration of TPMT was 0.05 mg/mL, a concentration chosen to minimize the concentration of recombinant-purified TPMT required for the assay. Each test inhibitor’s final concentration was 50 µM, 4-NBT had a final concentration of 200 µM, and the final concentration of SAM was 1 mM. The concentration of the test inhibitor was chosen to be 10× the K_m_ of 4-NBT. The concentration of 4-NBT was chosen to be 4× higher than the test inhibitor concentration to ensure that the absorbance signal from 4-NBT would be much greater than that from each inhibitor (Figure S3).

The absorbance at 411 nm was recorded while each test inhibitor was co-incubated with TPMT, 4-NBT, and SAM (Figure S4A). At the 1 h mark, the difference in absorbance at 411 nm, ΔAbs (411 nm), from the start of the incubation T0 to the end T60, was calculated (Figure 6B). We note that the 1 h mark was chosen arbitrarily; future iterations of this assay should pick a timepoint that is within the linear region of substrate disappearance [32]. By comparing the ∆Abs (411 nm) for each compound with the ∆Abs (411 nm) for the vehicle-control-treated sample, we determined that the only compound that did not significantly inhibit the methylation of 4-NBT was dopamine (Figure 6B). The negative control samples—the no-SAM and the boiled enzyme samples—had the smallest ∆Abs (411 nm), followed by olsalazine.

To compare the results from the absorbance assay with a conventional enzyme inhibition assay that utilizes mass spectrometry to monitor metabolite formation, TPMT was incubated with 6-MP, SAM, and a test inhibitor for 60 min at 37 °C and then quenched with acetonitrile containing the internal standard D_3_-6-MMP. During the reaction, each test inhibitor’s final concentration was 50 µM, 6-MP had a final concentration of 200 µM, and the final concentration of SAM was 1 mM. The K_m_ for 6-MP is around 300 µM [3]. We were concerned about the solubility of 6-MP at high concentrations, so the final concentration of 200 µM was chosen for this assay instead of a concentration above the K_m_ of 6-MP. We recorded the area under the curve (AUC) response for 6-MMP and D_3_-6-MMP with LC-MS/MS analysis following a 60 min enzyme incubation. The peak area ratio (PAR) for 6-MMP was calculated by dividing the AUC of 6-MMP by the AUC for D_3_-6-MMP. The PAR reported the relative level of 6-MMP for each test inhibitor and control-treated sample after a 60 min incubation. Similar to the absorbance assay, the only compound that did not significantly inhibit the methylation of 6-MP was dopamine (Figure 6 C). The negative control samples—no-SAM and the boiled enzyme samples—had the lowest level of 6-MMP, followed by olsalazine.

## 4. Discussion

Here, we present a new expression and purification method for producing pure active recombinant TPMT and a novel absorbance assay to screen compounds for TPMT inhibition. Previous purifications of TPMT were reported to be highly unstable; Woodson and Weinshilboum determined that, after the purification of TPMT from human kidneys, there was a 79% loss of activity within 24 h [1]. The TPMT purified using our new methodology was active and could methylate both 6-MP and 4-NBT, even after several rounds of freeze–thaw cycles (Figure S2). With this abundant source of active recombinant TPMT, we then optimized a TPMT activity assay that utilized absorbance change over time to report on the methylation of two TPMT substrates, 6-MP and 4-NBT.

Thiophenols and thiopurines will change color when their sulfur is alkylated or forms a disulfide bond. Previously, Cannon et al. developed an enzyme-coupled colorimetric assay to measure the amount of the methyltransferase active form of SAM stereoisomer in supplements. They accomplished this by measuring the absorbance change in 2-nitro-5-methylthiobenzoic acid (TNB) when it is methylated by TPMT [33]. We determined that 6-MP and a similar substrate to TNB, 4-NBT, could also be used to couple TPMT activity to a colorimetric assay (Figure 4B.I–B.III). We chose to use 4-NBT as a reporter substrate to screen for compounds that inhibit TPMT. 4-NBT has a relatively low published K_m_ of 6.5 µM, which allowed us to keep the substrate concentration relatively low so as not to saturate the absorbance detector.

Using 4-NBT as a probe substrate, we optimized a 96-well plate absorbance assay to rapidly screen for compounds that inhibit TPMT. A panel of 5 compounds was screened using this assay. Three of the compounds, balsalzide, sulfasalazine, and olsalazine, were benzoic acids known to inhibit TPMT [19,30,31]. One compound, dopamine, was known to not inhibit TPMT and the fifth compound, 5-sulfasalycilic acid, was chosen because it is a benzoic acid and expected to inhibit TPMT, but has not yet been tested for TPMT inhibition. 4-NBT has an absorbance maximum at 411 nm when it is a free thiol, but its maximum changes to 350 nm when it is methylated. We monitored for a decrease in absorbance at 411 nm over time. We chose to monitor 411 nm, which reports on parent disappearance, because the absorbance maximum for the metabolite had significant overlap with several of the test compounds (Figure S3). TPMT was reacted with 4-NBT, SAM, and a test compound for 1 h. At the 1 h mark, the difference in absorbance at 411 nm, ΔAbs (411 nm), from the start of the incubation T0 to the end T60, was calculated (Figure 4). All compounds tested except for dopamine had a significantly smaller ΔAbs (411 nm) value than vehicle-control-treated samples. We confirmed these results by incubating each compound with TPMT, SAM, and 6-MP, and then used mass spectrometry to measure 6-MP methylation. Identical to our absorbance assay results, four compounds other than dopamine caused a reduction in substrate methylation. These results confirm that an absorbance assay using 4-NBT as a probe substrate is suitable to identify compounds that inhibit TPMT.

Plate-based absorbance assays are advantageous over other analytical methods such as LC-MS/MS because they have a lower cost and can be adapted for high-throughput screening. It is crucial that compounds of interest do not overlap with both the 4-NBT parent absorbance maximum and its methyl metabolite’s absorbance maximum. The absorbance assay reported here could be used as a low-cost and high-throughput way to screen many drugs on the market or in development for TPMT inhibition. In a 2021 study, Lin et al. concluded that patients receiving azathioprine co-therapy with mesalazine for irritable bowel syndrome had a clearance rate of thioguanine nucleotides that was 0.802 times lower than that in patients who were not receiving mesalazine [34]. There is a clear link between TPMT inhibition and thiopurine drug safety and effectiveness. The FDA now recommends caution for the concomitant use of azathioprine and benzoic acids like mesalazine. It is interesting to speculate whether benzoic acids would have been identified as causing clinically relevant interactions without their prior identification as TPMT inhibitors in vitro. Understanding the drug interaction potential for all drugs that may be administered concomitantly with thiopurines, not only those with benzoic acid moieties, will ultimately improve clinical decision making when developing thiopurine-based treatment plans.

## 5. Conclusions

We have developed a novel improved method for producing pure and highly active recombinant TPMT. We then used the purified TPMT to develop a novel absorbance-based assay that can be used to screen compounds for TPMT inhibition. This assay monitors the absorbance change in TPMT substrates as they are methylated. We used this assay to screen for compounds that, when added to the methylation reactions, inhibit recombinant-purified TPMT. The absorbance assay performed just as well as a conventional mass-spec-based assay at identifying compounds that inhibit TPMT but with fraction of the time and cost. We hope that the absorbance assay and new TPMT purification method will be used to expand the number of compounds screened for TPMT inhibition. An improved understanding of the factors contributing to variable TPMT activity across individuals will ultimately improve clinical practice when developing thiopurine-based treatment plans.

## Data Availability

The original contributions presented in this study are included in the article and Supplementary Material. Further inquiries can be directed to the corresponding author.

## Supplementary Materials

The following supporting information can be downloaded at: https://www.mdpi.com/article/10.3390/metabo14120715/s1. Figure S1: SDS-PAGE Coomassie stain of purified recombinant TPMT purification components; Figure S2: Real-time monitoring of 4-NBT disappearance and 4-NBMT appearance after TPMT is subjected to an increasing number of freeze-thaw cycles; Figure S3: Overlayed absorbance scans, from 300 to 550 nm, of 4-NBT and dopamine, balsalazide, sulfasalazine, 5-sulfosalicylic acid, and olsalazine; Figure S4: Real-time monitoring of 4-NBT disappearance in the presence of different test inhibitors.
