# Supplementary material for: A Rapid and Reliable Absorbance Assay to Identify Drug–Drug Interactions with Thiopurine Drugs
Source: Metabolites. 2024 Dec 19;14(12):715. doi: 10.3390/metabo14120715 (PMC11679344; doi:10.3390/metabo14120715)
Supplement: Supplementary file 1 [file metabolites-14-00715-s001.zip › metabolites-3348944-supplementary.pdf]

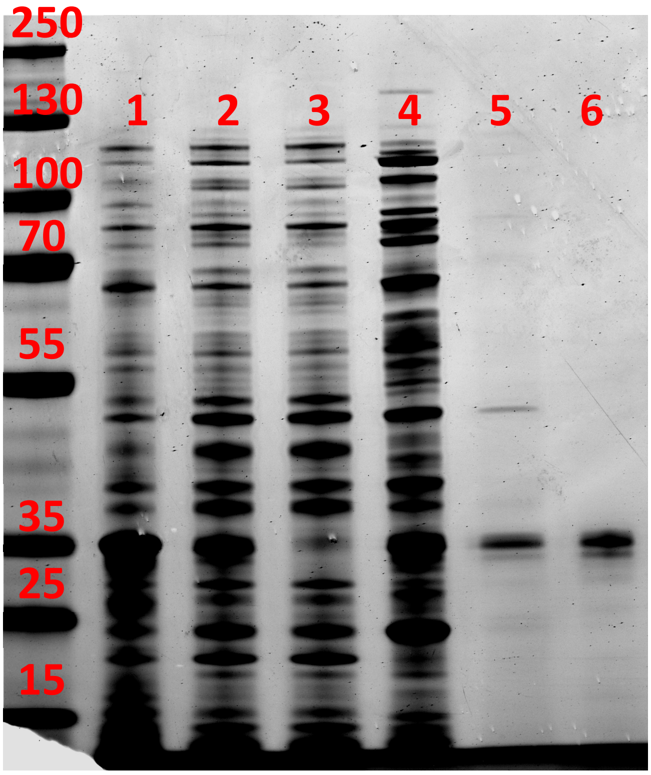

Figure S1. SDS-PAGE Coomassie stain of purified recombinant TPMT purification components. Protein amount lower than figure 6 to show detail in lane 6. Lane Order: homogenate (1), lysate (2), lysate post Ni column load (3), wash 1 (4), wash 2 (5), and eluent (6).

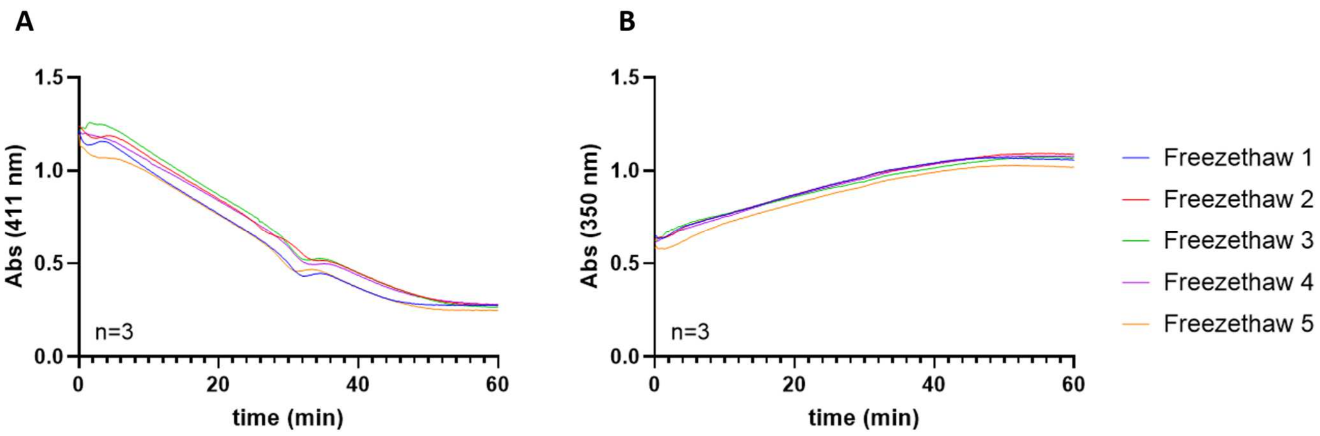

Figure S2. Real-time monitoring of 4-NBT disappearance (A) and 4-NBMT appearance (B) after TPMT is subjected to an increasing number of freeze-thaw cycles. Data are presented as the mean.

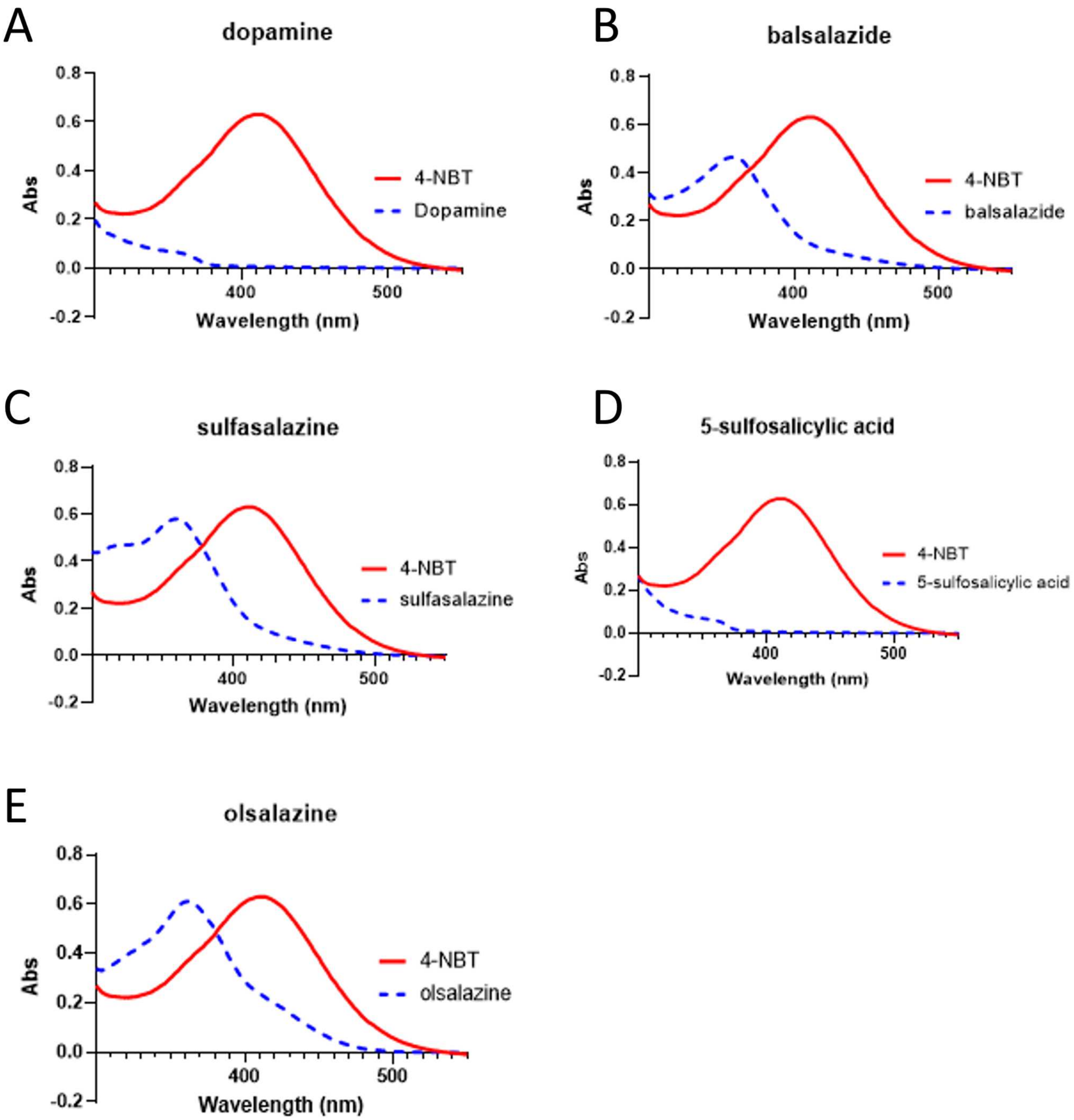

Figure S3. Overlayed absorbance scans, from 300 to 550 nm, of 4-NBT and dopamine (A), balsalazide (B), sulfasalazine (C), 5-sulfosalicylic acid (D), and olsalazine (E). Data are presented as the mean, n=3.

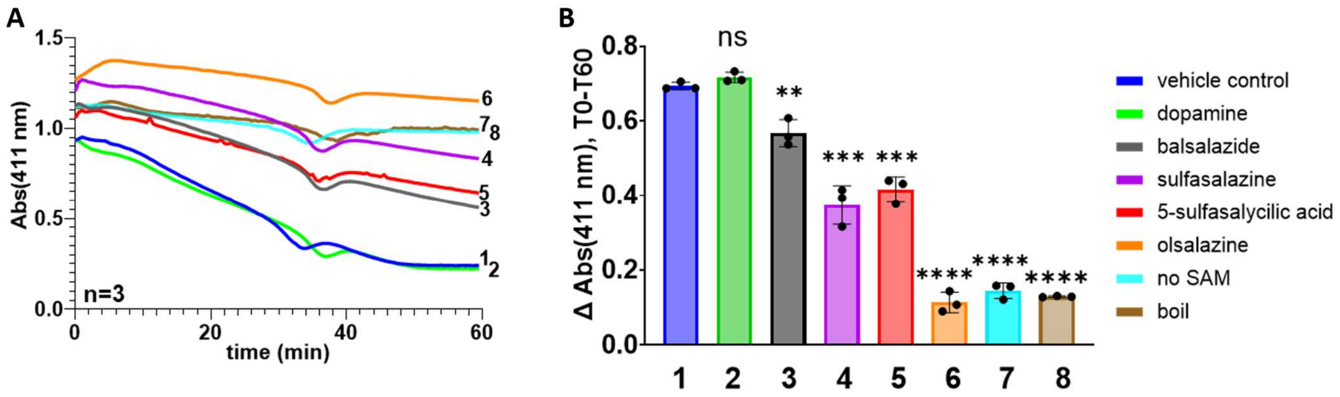

Figure S4. Real-time monitoring of 4-NBT disappearance in the presence of different test inhibitors (A). The difference in absorbance at 411 nm from T0 to T60 (B) in the presence of various test inactivators. Data in A are presented as the mean. Data in B are presented as the mean ± S.D. ****P < 0.0001; ***P < 0.001; **P < 0.01.
